# Optical neuroimaging and neurostimulation in surgical training and assessment: A state-of-the-art review

**DOI:** 10.3389/fnrgo.2023.1142182

**Published:** 2023-03-20

**Authors:** Mary Goble, Virginia Caddick, Ronak Patel, Hemel Modi, Ara Darzi, Felipe Orihuela-Espina, Daniel R. Leff

**Affiliations:** Department of Surgery and Cancer, Imperial College London, London, United Kingdom

**Keywords:** neuroergonomic, functional near-infrared spectroscopy, surgical training, neurostimulation, neuroimaging, neuromonitoring

## Abstract

**Introduction:**

Functional near-infrared spectroscopy (fNIRS) is a non-invasive optical neuroimaging technique used to assess surgeons' brain function. The aim of this narrative review is to outline the effect of expertise, stress, surgical technology, and neurostimulation on surgeons' neural activation patterns, and highlight key progress areas required in surgical neuroergonomics to modulate training and performance.

**Methods:**

A literature search of PubMed and Embase was conducted to identify neuroimaging studies using fNIRS and neurostimulation in surgeons performing simulated tasks.

**Results:**

Novice surgeons exhibit greater haemodynamic responses across the pre-frontal cortex than experts during simple surgical tasks, whilst expert surgical performance is characterized by relative prefrontal attenuation and upregulation of activation foci across other regions such as the supplementary motor area. The association between PFC activation and mental workload follows an inverted-U shaped curve, activation increasing then attenuating past a critical inflection point at which demands outstrip cognitive capacity Neuroimages are sensitive to the impact of laparoscopic and robotic tools on cognitive workload, helping inform the development of training programs which target neural learning curves. FNIRS differs in comparison to current tools to assess proficiency by depicting a cognitive state during surgery, enabling the development of cognitive benchmarks of expertise. Finally, neurostimulation using transcranial direct-current-stimulation may accelerate skill acquisition and enhance technical performance.

**Conclusion:**

FNIRS can inform the development of surgical training programs which modulate stress responses, cognitive learning curves, and motor skill performance. Improved data processing with machine learning offers the possibility of live feedback regarding surgeons' cognitive states during operative procedures.

## 1. Introduction

Neuroergonomics is concerned with the study of brain behavior under naturalist conditions. It encompasses the application of neuroscience, and the biological substrates that underlie brain function, to ergonomics. “Surgical neuroergonomics”, the convergence of two disciplines in the surgical setting, seeks to understand the cognitive processes that underpin operative performance, and inform the design of safer operating systems and more robust training paradigms. Various neuromonitoring techniques have been used to increase the understanding of neural processes that underpin task acquisition in surgery. Examples of such modalities include functional magnetic resonance imaging (fMRI), positron emission topography (PET), electroencephalography (EEG), and functional near-infrared spectrometry (fNIRS).

**Figure 1:**
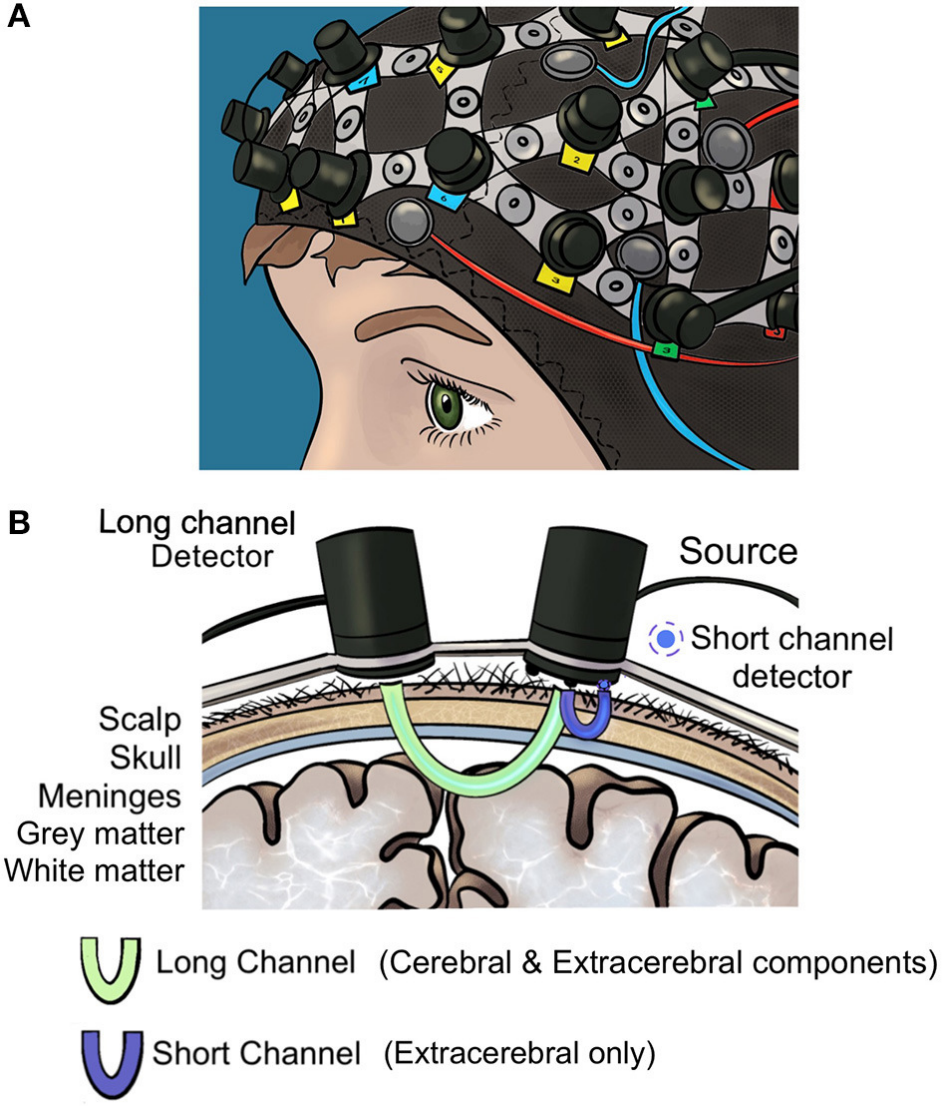
**(A)** Optical neuroimaging cap **(B)** long and short fNIRS detector channels. Short detector channels measure confounding extracerebral signal whilst long channels measure extracerebral and cerebral signals.

Of these, the optical neuroimaging modality fNIRS is the most frequently used technique for surgical neuroergonomic applications (Patel et al., 2020; Hannah et al., 2022) as it is safe, radiation free, comparatively inexpensive and enables surgeons to operate freely in naturalistic settings (Modi et al., 2017a; Chen et al., 2020). In fNIRS, near-infrared light is radiated to the brain *via* light sources and absorbed by oxygenated (HbO2) and deoxygenated (HHb) hemoglobin in the cerebral vasculature (Figure 1). The Modified Beer-Lambert Law estimates the optical parameters (absorption coefficient) from which the brain oxygenation is later derived. Areas of increased cerebral activity correlate with an increase in oxygen requirement, however there are regional (e.g., extracerebral) confounders (Erdogan et al., 2014; Scholkmann et al., 2022) which should be recorded to increase the accuracy of fNIRS signals (Scholkmann et al., 2022). Conventionally, fNIRS uses “long channels”, where light emitters are placed 3 cm away from detectors on the scalp. “Short channels” placed 8–10 mm away from the light source demonstrate strong bias toward extracerebral signals (scalp, skull and meninges); the addition of these channels enables regression of confounding haemodynamic activity (Strangman et al., 2013; Brigadoi and Cooper, 2015; Caldwell et al., 2016; Reddy et al., 2021). Systemic factors (e.g., heart rate, arterial CO_2_) likewise act as confounders in multiple ways, illustrated by the vasodilatory effect of CO_2_ which modulates neurovascular coupling, or noise generated from heart rate variations (Lindauer et al., 2010; Kirilina et al., 2012; Caldwell et al., 2016; Scholkmann et al., 2022). Recording systemic responses using instruments such as capnographs or plethysmographs allows for regression of these effects out of fNIRS signals.

Whilst neural patterns of activation in surgeons related to stress and expertise have previously been reviewed systematically (Arora et al., 2010; Modi et al., 2017b; Hannah et al., 2022), studies conducted since (Crewther et al., 2016; Singh et al., 2018; Modi et al., 2020) merit inclusion for an up-to-date understanding of the progress made in the field of surgical neuroergonomics. In particular, prior reviews narrowed their scope, omitting recent developments in neuroimages of stress and performance enhancement such as robotic assistance or neuroaugmentation (e.g., neurostimulation). To address these gaps, we conducted a narrative review of literature across key areas including surgical skill assessment and proficiency, workload and stress and performance enhancement either through external or robotic assistance or neurostimulation. Across these domains we specifically aimed to determine whether neuroimages can reliably classify surgical skill; whether there are neural signatures of high mental workload; and aimed to characterize the impact of performance enhancing interventions on neural responses in surgeons. Finally, we aimed to highlight key progress areas required in neuroergonomics to implement live cognitive feedback in the theater environment, including trends in data collection and processing. Findings are summarized in Table 1.

**Table 1:** Summary of current understanding, research gaps and future applications of neuromonitoring and neurostimulation to surgical training.

**Current understanding**	**Knowledge gaps: Research priorities**	**Future applications to surgical training**
fNIRS data accuracy is improved by measurement of regional and systemic blood flow	How can data processing be optimized - AI/machine learning? How can data be collected from complex, real operating theatre environments?	Live fNIRS in theater—instant training feedback and modulation of behavior
Experts exhibit less activation in PFC and SMA/M1/PMA than novices during motor skill	What is the neural benchmark of expertise in motor skill and task planning?	Neural signature patterns to demonstrate proficiency
Decline in performance due to stress is linked to decreased activation in PFC	How do different types of stressors affect cognitive activation and brain networks? Does expertise effect performance under stress?	Understanding of stress response in order to prevent performance deterioration Evaluating stress-reducing interventions
Robotic training tools lead to increased PFC activation compared to laparoscopic	How does robotic surgery vs. laparoscopic surgery impact rate of skill acquisition and mastery?	Training tools developed to enhance cognitive response
Direct stimulation enhances motor performance in surgical trainees	What are the long term benefits and risks of tDCS? What is the effect of tDCS on patient outcomes?	Use of tDCS to improve rate of learning

## 2. Surgical skills assessment and proficiency

The versatility and accessibility of fNIRS has enabled the study of cognitive activation patterns with surgical tasks and training, exploring the effects of task complexity and expertise. Areas implicated in motor tasks include the prefrontal cortex (PFC), supplementary motor area (SMA), primary motor cortex (M1), and cerebellum (Modi et al., 2017b; Hannah et al., 2022). The PFC can be further subdivided into the dorsolateral (DLPFC), ventrolateral (VLPFC) and medial-frontopolar subregions. fNIRS array design and channel sensitivity distribution can optimize spatial resolution of the underlying macroanatomy, to better understand specific functional localization (Walia et al., 2021). In simple bimanual tasks such as knot-tying, novices exhibit greater DLPFC activation than experts, this effect diminishes with practice and improved performance (Shetty et al., 2016; Modi et al., 2017b; Izzetoglu et al., 2021; Hannah et al., 2022). Izzetoglu et al. (2021) note deactivation specifically in the left DLPFC. The DLPFC is understood to subserve executive functions: working memory and attention, task-planning and cognitive flexibility (Carlén, 2017). For more complex tasks, such as laparoscopic or robotic suturing, the training period required to first observe PFC excitation and indeed subsequent reduction in PFC activation is substantially longer (Walia et al., 2022). Ohuchida et al. (2009) observed minimal PFC activation in those naïve to laparoscopic suturing with subsequent practice-related PFC attenuation occurring over a protracted time interval. Shetty et al. (2016) describe persistent PFC activation in trainees despite a week of practice and technical performance improvement; similarly, Walia et al. (2022) describe right DLPFC activation in an expert group. Only after years of practice and regular task execution was PFC attenuation observed in expert consultants (Shetty et al., 2016).

Expertise is also characterized by re-organization and redistribution of activation across other motor regions of the cortex; bi-manually skilled experts recruit regions involved in action observation (ventral premotor cortex), motor planning (posterior parietal cortex) and action execution (M1) in order to improve task efficiency (Yang, 2015). Increased supplementary motor area (SMA) and primary motor cortex (M1) activation having been demonstrated to be positively correlated with expertise (Nemani et al., 2018; Hannah et al., 2022). During surgical task planning, motor and parietal regions demonstrate greater activation in experienced surgeons compared to novice students, the latter who exhibit greater bilateral DL, VL and medial PFC activation when making decisions compared to experts, who gain in economy of movement and precision (Bann et al., 2003). Ventromedial activation is also prominent in novices and has been shown to be absent in experts (Leff et al., 2017). The cerebellum's function in motor coordination is reflected in optical neuroimaging surgical data: in laparoscopic tasks, experts demonstrate increased cerebellar activation, whilst novices display attenuated cerebellar activation (Duty et al., 2012).

Correlation between haemodynamic responses across different brain regions demonstrates disparate ‘functional connectivity' between novice and expert surgeons (Nemani et al., 2021; Kamat et al., 2022). For example, Andreu-Perez et al. (2016) demonstrated that during complex bimanual surgical co-ordination tasks, functional connectivity data predicted operator skill level with good accuracy, experts displaying greater motor connectivity whilst novices demonstrated stronger connectivity in prefrontal and premotor regions. In assessing connectivity, the application of fuzzy cognitive maps, capable of describing non-linear relationships and changing connectivity by delivering a graphical representation of a given system, is a complementary technique based on Kosko's causal definition to add to other current methods based on Granger's causal definition, including dynamic causal modeling (Kiani et al., 2022).

Understanding cognitive patterns associated with expertise allows trainers to define neural proficiency, for example *via* a defined threshold of changes that accompany skill proficiency, which could be spatial distribution of activations, or magnitude of activation in certain regions of interest. Trainees engage in a substantial amount of simulated and virtual learning due to reduced training opportunities and a concern for patient safety, hence a need to ensure simulated and virtual training bestow the same level of skill than physical training (Seymour et al., 2002; Aggarwal et al., 2007; Hogle et al., 2009). Currently, surgical proficiency is assessed against benchmarks such as time taken to perform a skill, numbers of errors made, or subjective rater assessment (Seymour et al., 2002; Aggarwal et al., 2007; Munz et al., 2007). Time-based metrics have been shown to poorly classify trainee expertise (Nemani et al., 2019), whereas fNIRS has been shown to at least match, if not surpass, current metrics in being able to reliably distinguish levels of expertise between trainees, in turn confirming the value of simulated and virtual training (Nemani et al., 2018, 2019; Gao et al., 2021b; Hannah et al., 2022). As highlighted by Nemani et al. (2019) current measures of performance of laparoscopic tasks (such as time to complete task) more frequently misclassify level of motor skill compared to fNIRS.

The singularity of fNIRS may however lie its potential to set a benchmark of neural proficiency for complex, time-consuming tasks, which cannot be assessed with simple time-based measures and are difficult to evaluate beyond patient outcomes and complication rates. To enable this application of optical neuroimaging, it must develop the capacity to integrate and process large amounts of data in real time during prolonged periods, mapping evolving cognitive function throughout an operation to create a benchmark of expert patterns of activation.

## 3. Cognitive workload and stress

Optical neuroimaging can provide an objective measure of cognitive workload. Cognitive workload theory describes intrinsic (inherent to the task), extraneous (the context in which the task is situated), and germane (the processing ability of the subject) cognitive load (Sweller, 2011). Stress adds to extraneous workload and has independently been found to impair surgical performance, affecting both technical and non-technical skills such as communication skills and decision-making (Arora et al., 2010; Crewther et al., 2016; Modi et al., 2018; Singh et al., 2018). Current understanding suggests an inverted-U shape association between PFC activation and mental workload, with activation increasing in-line with workload demands, before attenuating once it passes a critical inflection point at which demands outstrip cognitive capacity (Ossewaarde et al., 2011; Modi et al., 2018; Singh et al., 2018). Modi et al. (2018) demonstrated performance degradation during timed conditions, or temporal stress, associated with relative PFC attenuation. In a subsequent study, they integrated a cognitive stress component (e.g., decision-making) and demonstrated a cumulative effect with temporal stress, PFC deactivation being greatest in temporally and cognitively stressed scenarios (Modi et al., 2020). Furthermore, between-subject variation in behavioral responses to stress may have a basis in the quantitative difference in PFC deactivation: trainees who maintain technical performance under stressful conditions demonstrate increased haemodynamic responses (change in HbO2) in the bilateral ventrolateral PFC and left dorsomedial PFC as compared to trainees whose performance degrades (Modi et al., 2019).

The effect of expertise on stress responses detected by fNIRS is unclear. An improvement in performance under stress conditions has been correlated with increased expertise in certain studies (Modi et al., 2018), whereas others found no association between performance deterioration under stress conditions and expertise (Modi et al., 2019). Research is required to further clarify the relationship between expertise, stress and cortical neuroimage data, which ultimately may indicate a need for a measure of stress resilience independent from expertise. There may be a role for fNIRS not only as a measure for technical proficiency, but also a measure of the neural stress response, which can be targeted by way of individualized surgical training.

The value of measuring neural stress responses may be debated: if neural stress patterns are confined to the cerebral response and do not translate into a deterioration in practice, then what relevance does detecting changes in neural responses have clinically? Conversely, if we are able to correlate increasing stress with decline in performance, then measure the performance decline instead of the neural response. Importantly, neural responses may provide a more accurate measure of stress than physiological measures such as heart rate variability or subjective measures of workload (Modi et al., 2019). Optical neuroimaging may also have a role in assessing the effectiveness of stress reducing interventions such as noise reduction (Engelmann et al., 2014). In addition, establishing a clear mechanism between the onset of stress and neural responses leading to deterioration in performance potentially facilitates interception of stress before progression to performance deterioration, targeting the process before it leads to deleterious effects or clinical harm.

## 4. Optimizing training tools

Modern operating tools such as robotic or laparoscopic systems promise to ease the operative burden for trainees and experts. fNIRS has a role in evaluating these systems, by measuring the effect they have on surgeons' brain activation. The superiority of robotic-assisted vs. laparoscopic training is argued for in the literature, citing improved ergonomics, intuitiveness, instrument manipulation and learning curve attenuation (Chandra et al., 2010; Stefanidis et al., 2010; Singh et al., 2018). Indeed, when compared to laparoscopic surgery, surgeons perform better on robotic platforms, despite being less experienced in using them, and exhibit greater PFC activation (Singh et al., 2018).

The advent of robotic systems is also associated with integrated technological aids such as gaze-enhanced or gaze-assisted learning (GEL), a technique proposed to improve motor skills during robotic surgery by constraining the movements of the robot to limit its actions to areas being “gazed” at by the surgeon (Mylonas et al., 2012). This is based on the postulate that as expertise develops, the surgeon's eye movements focus on the target area. An eye tracking device establishes the target area and incorporates this into movement restrictions placed on the robot, which means the surgeon encounters mild resistance if deviating away from the target area. The resistance can easily be overridden by the surgeon who maintains full control. Compared to trainees using robotic systems with no learning aids, GEL leads to greater PFC activation in novice trainees (Mylonas et al., 2012), followed by rapid attenuation of PFC after few practice sessions (James et al., 2013).

At equal expertise level, greater activation of PFC is understood to correlate with increased focus and attention on the task at hand, the cognitive learning curve for motor skills demonstrating first an increase in activation in PFC, M1, SMA, and premotor area (PMA) (Ungerleider et al., 2002; Luft and Buitrago, 2005), followed by a decrease in activation in PFC, SMA and PMA and reorganization of M1 once the task is mastered allowing trainees to lower the intensity of attention directed toward skill performance. What remains to be uncovered is whether short term increase in PFC activation does indeed accelerate the learning curve, or whether it represents the need for increased attention throughout the learning process. Further data collection, including in longitudinal studies of trainees, is necessary to understand the differences between laparoscopic and robotic systems on cognitive performance and the benefits of technological aids. These have the potential to “outsource” processing of data to the machine, and reduce cognitive load, provided it does not impair the neural process of learning.

## 5. Neurostimulation

Direct visualization of cognitive processes which occur during training opens the possibility of targeting these processes with direct cognitive stimulation. Neurostimulation may optimize cognitive responses to surgical training, improving the quality and speed of skill acquisition (Dissanayaka et al., 2017; Biabani et al., 2018; Ciechanski et al., 2018; Patel et al., 2019, 2021; Hung et al., 2021). Transcranial direct-current stimulation (tDCS) is a safe, non-invasive neurostimulation technique (Paulus, 2011) which sustains low-amplitude current (typically < 2 mA) through the brain and modulates neuronal excitability and plasticity. Its intensity is too low to generate neuronal excitability de-novo (Paulus, 2011; Morya et al., 2019). Improvement in motor task performance is conferred safely through tDCS (Bikson et al., 2016; Patel et al., 2019, 2021; Gao et al., 2021a; Hung et al., 2021) along with the prevention of stress induced working memory impairment, which suggests new applications for neuroenhancement (Bogdanov and Schwabe, 2016). Further clarification is still needed regarding the types of skill neurostimulation interventions may enhance as there may be some task-dependent effect on the strength of improvement of learning; in other words, certain tasks may be more improved than others through the use of tDCS. Similarly, the long-term effects on motor retention may also be task specific (Buch et al., 2017).

Technical considerations such as the configuration of the tDCS setup, including the intensity of stimulation delivered and the areas of the cortex to which the stimulation is applied to, should be an area of focus for future study (Batsikadze et al., 2013; Morya et al., 2019). Miniaturization of the neurostimulation technology will facilitate integration into training settings or the live theater. This may be realized through the advent of high definition tDCS which shrinks the large electrode pads conventionally applied to the cortex to targeted electrodes applied to specific regions of the brain (Kuo et al., 2013). There is so far limited data regarding the superiority of HD-tDCS compared to conventional tDCS, outlining another area for further study.

These knowledge gaps notwithstanding, there are broader questions which must be answered prior to the advent of neurostimulation in the training or live theater environment. Current data indicates that tDCS is safe, however there no studies investigating the long-term effect of stimulation on surgeons' motor skills. In addition, the added benefit of using neurostimulation must be based on enough evidence to warrant not only the adverse effects, but also the technical challenges of deploying devices such as fNIRS or tDCS in real operating theater environments. Currently, demonstrated effect sizes regarding the advantage of tDCS on surgical skill may not be large enough to justify this, however more data is required to reach a reliable conclusion (Ciechanski et al., 2019). Other factors must also be taken into consideration, such as the opinions of trainees and patients. Whilst data suggests they are supportive of neuroenhancement (Patel et al., 2021), trainees may be reluctant to devote time, money, and effort to new technologies before there is a strong evidence base to prove that these interventions improve outcomes.

## 6. Discussion

### 6.1. Future challenges: Toward real-time performance modulation

Currently, the use of optical neuroimaging in the surgical community is aligned with the historical use of neuroimaging broadly, which is to describe neural response patterns. This has enabled the identification of differential PFC, SMA, M1, and PMA activation, and neural connectivity, in novices and experts in various contexts, including stress conditions. This knowledge can serve to define cognitive benchmarks of expertise, ensuring high standards of objective performance assessment in surgical training (Nemani et al., 2019).

Whilst fNIRS is undoubtably a powerful and accurate observational tool, its added value in surgical training and assessment lies in its potential to not only describe but modulate stress responses, cognitive learning curves, and motor skill performance. Identifying the critical inflection point of the hypothesized inverted “U-curve”, where excess workload causes PFC attenuation, predicting performance deterioration, would allow timely intervention in the moment of motor skill performance, prompting a change in behavior or environment. Over a more prolonged timeframe, frequent visualization of trainee cognitive learning curves allows for direct targeting of specific skills, individualizing the type and quantity of practice necessary to align with the cognitive benchmarks of proficiency previously established. Behavior can be modulated *via* conscious feedback, the surgeon being made aware of their cognitive state and prompted to change their mindset or movements; it can also be modulated *via* aforementioned neurostimulatory modalities.

The cornerstone of bringing optical neuroimaging into the operating theater is, however, the successful development of more accurate, real-time, data processing. Two main components of data processing are regression of physiological parameters and motion artifact removal, both which may act as confounders for true cerebral haemodynamic activity. Recent experience with short channel regression and increasingly with systemic physiology measures have enabled the development of models predicting the impact of extra-cerebral factors on haemodynamic activity. Machine learning algorithms utilize such models to improve data quality, bypassing the need for manual correction. In addition to improving the efficiency of processing, combined regression of systemic physiology measurements and short channel fNIRS also provides superior differentiation of the confounders (Scholkmann et al., 2022). Combined systemic-physiology-augmented-fNIRS (SPA-fNIRS) data analysis methods include generalized linear model (GLM) regression and time-embedded-canonical-correlation analysis (Ortega-Martinez et al., 2022), or wavelet transformation (von Lühmann et al., 2020). Deep learning, a type of machine learning, may provide fast, accurate removal of motion artifact within fNIRS data, with noise features being learnt by software removing the need for assumptions regarding the noise characteristics to be described by the research team (Gao et al., 2022). Whilst machine learning is helpful adjunct, it has yet to prove superiority in improving data quality or accurate detection of artifacts as compared to hand-resolved models.

The limitations of diversifying fNIRS research methodology must be recognized, as the variety of hardware and acquisition parameters, lack of standardization of optode array placement and registration, nomenclature of neuroanatomy and data-processing/regression of confounders make literature interpretation and comparison challenging (Hocke et al., 2018; Nemani et al., 2018; Yücel et al., 2021). Signal quality is subject to distortion by environmental and device noise, poor optode coupling, subject motion artifact, and optical interference by coarse/pigmented hair. Yücel outlines consensus guidelines for methodological reporting in an effort to circumvent such challenges, improving interpretation, reliability and reproducibility of studies (Yücel et al., 2021).

## 7. Conclusion

FNIRS is a non-invasive optical neuroimaging technique which has been widely used to assess surgeons' brain function. It has described the cognitive activation patterns of novice surgeons, who exhibit greater haemodynamic responses across the pre-frontal cortex than experts when performing simple surgical tasks. Expert surgical performance is characterized by relative prefrontal attenuation and upregulation of activation foci across other motor regions such as the supplementary motor area. In addition to localized changes in activation, greater connectivity between motor areas is noted in experienced surgeons, whilst novices demonstrate stronger connectivity in prefrontal and premotor regions. The neural effect of stress has been hypothesized to follow an inverted “U-shape”, with critical workload leading to a decrease in pre-frontal activation and deterioration in performance; trainees who maintain technical performance under stress demonstrate less PFC attenuation. Neural correlates of expertise and optimal performance suggest optical neuroimaging is a suitable tool to assess proficiency since neuroimages perform comparably, if not better, than current measures of aptitude, and could depict a cognitive state during surgery leading to the development of cognitive benchmarks of expertise. Beyond the observational abilities of optical neuroimaging, it has the potential to observe identify performance deterioration and trigger a reaction, individualizing training programs to optimize the learning curve, and by direct neurostimulation to accelerate skill acquisition and enhance performance. These future applications of optical neuroimaging and neurostimulation in the live operative environment rely upon the development of improved data processing.

## Author contributions

MG, VC, DL, and RP contributed to conception and design of the work and drafted the work. DL, RP, FO-E, and HM revised it critically for important intellectual content. MG, VC, RP, HM, AD, FO-E, and DL provide approval for publication of the content and agree to be accountable for all aspects of the work in ensuring that questions related to the accuracy or integrity of any part of the work are appropriately investigated and resolved. All authors contributed to the article and approved the submitted version.
